# The m^6^A RNA methylation regulates oncogenic signaling pathways driving cell malignant transformation and carcinogenesis

**DOI:** 10.1186/s12943-021-01356-0

**Published:** 2021-04-04

**Authors:** Mohammad Burhan Uddin, Zhishan Wang, Chengfeng Yang

**Affiliations:** Division of Cancer Biology, Department of Medicine, MetroHealth Medical Center, Case Western Reserve University School of Medicine, Cleveland, OH 44109 USA

**Keywords:** m^6^A, Epitranscriptomics, Cell transformation, Carcinogenesis, Signal transduction

## Abstract

The m^6^A RNA methylation is the most prevalent internal modification in mammalian mRNAs which plays critical biological roles by regulating vital cellular processes. Dysregulations of the m^6^A modification due to aberrant expression of its regulatory proteins are frequently observed in many pathological conditions, particularly in cancer. Normal cells undergo malignant transformation via activation or modulation of different oncogenic signaling pathways through complex mechanisms. Accumulating evidence showing regulation of oncogenic signaling pathways at the epitranscriptomic level has added an extra layer of the complexity. In particular, recent studies demonstrated that, in many types of cancers various oncogenic signaling pathways are modulated by the m^6^A modification in the target mRNAs as well as noncoding RNA transcripts. m^6^A modifications in these RNA molecules control their fate and metabolism by regulating their stability, translation or subcellular localizations. In this review we discussed recent exciting studies on oncogenic signaling pathways that are modulated by the m^6^A RNA modification and/or their regulators in cancer and provided perspectives for further studies. The regulation of oncogenic signaling pathways by the m^6^A modification and its regulators also render them as potential druggable targets for the treatment of cancer.

## Background

Carcinogenesis is a complex process stemming from genetic and epigenetic changes in a specific cell population. Epigenetic changes that are conventionally known for differential gene expression pattern leading to alternative phenotypic traits involve- altered DNA methylation, modification of histone or remodeling of chromatin structure. Recently, the RNA posttranscriptional modification has emerged as another epigenetic gene expression regulatory mechanism. The ubiquitous presence of modified nucleotides in all kinds of RNA transcripts (both coding and non-coding) has given rise to a completely new concept named ‘Epithanscriptomics’ [1, 2].

Among all the modifications m^6^A is the most abundant internal modification of mRNAs and long non-coding RNAs (lncRNAs). In mRNAs, m^6^A sites are highly enriched in 5′-untranslated regions (5′-UTRs), near the stop codons, in the 3′-untranslated regions (3′-UTRs) and within long internal exons. m^6^A most frequently occurs in the consensus sequence RRACH (R = G/A/U, A = m^6^A, H=U/A/C) and such sequence occurs about every 85 nucleotides. Thus, the frequency of m^6^A has been estimated as 3–5 sites per mRNA transcript and 0.1–0.4% of all adenosine nucleotides in mammalian mRNAs [3, 4]. Due to vital functions of m^6^A modification that are observed in multiple cellular processes, dysregulation of the modification and its regulators have been implicated in various human diseases, especially in different types of cancers as reviewed in recent literatures [3, 5]. Here we discussed recent findings that demonstrate the role of m^6^A in modulating cellular oncogenic signaling pathways leading to carcinogenesis.

## Regulation of m^6^A modification

**Fig. 1:**
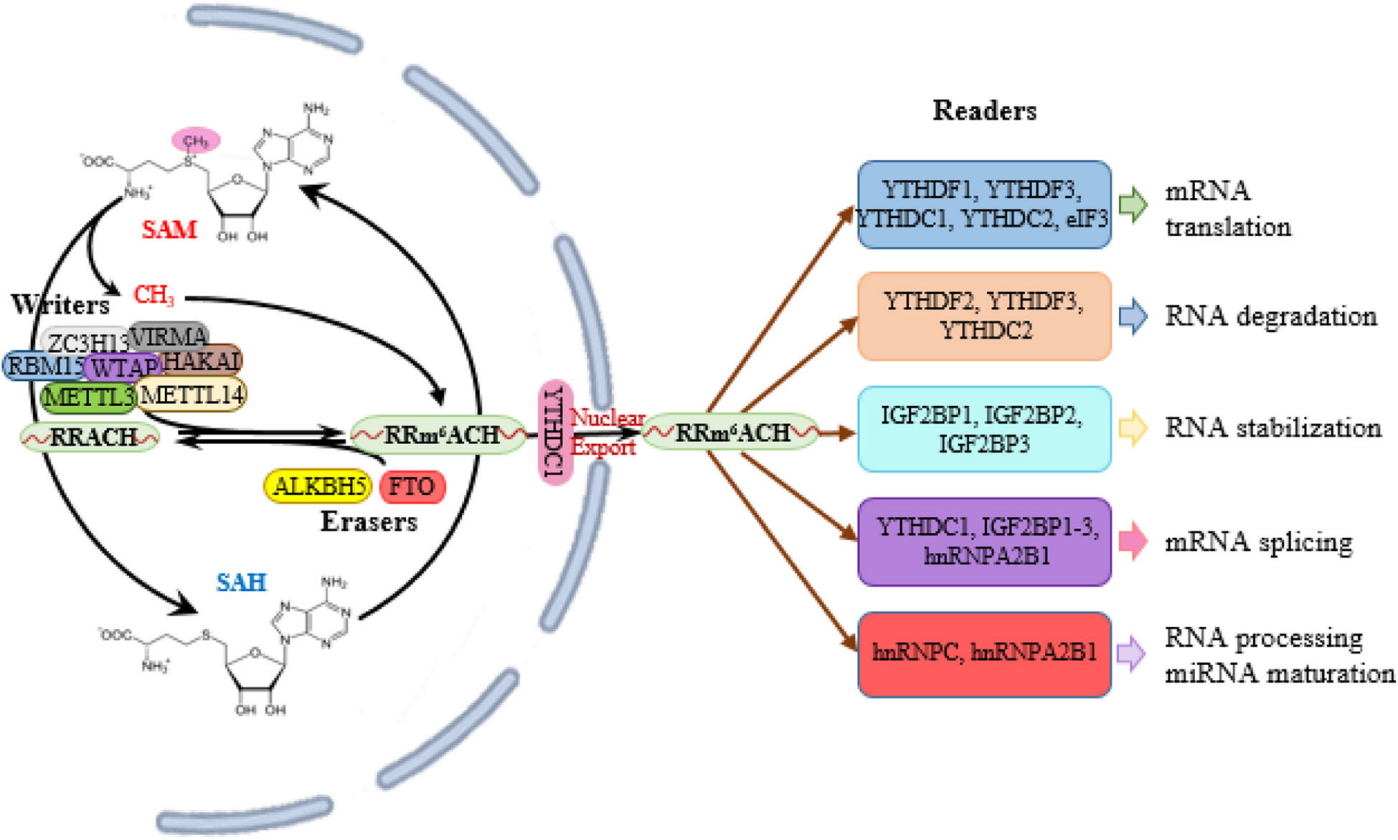
Regulation of the m^6^A modification. The ‘Writers’ catalyze the transfer of methyl group from SAM to the A to introduce m^6^A methylation in their target transcripts while the ‘Erasers’ remove the methyl group from m^6^A. The ‘Reader’ proteins recognize the m^6^A methylation marks in the modified transcripts and determine their fate. SAM, S-Adenosyl methionine; SAH, S-Adenosyl homocysteine; METTL3, Methyl transferase-like 3; METTL14, Methyltransferase-like 14; WTAP, Wilms tumour 1-associated protein; VIRMA, Vir-like m^6^A methyl transferase associated protein; ZC3H13, Zinc finger CCCH domain-containing protein 13; RBM15, RNA-binding motif protein 15; FTO, Fat mass and obesity-associated protein; ALKBH5, AlkB homologue5; YTH, YT521B homology; hnRNPs, Heterogenous ribonucleoproteins; IGF2BP, Insulin-like growth factor 2 mRNA-binding protein

The m^6^A modification is a highly dynamic and reversible process that involves enzymes responsible for- installing the modification called ‘writers’, removal of methylation called ‘erasers’ and recognition of the modification called ‘readers’ (Fig. 1). Members of every classes of regulators work together in a concerted manner to maintain a steady-state balance of m^6^A level inside the cell.

## Writers

The m^6^A writers are methyltransferases that catalyze the transfer of a methyl group from S-adenosylmethionine (SAM) to the N-6 position of adenosine (A). The methyltransferases were first identified as a multicomponent complex in 1994 [6]. Methyltransferase like-3 (METTL3) and its homolog METTL14 were later identified as two components of the complex [7]. Shortly after the discovery of METTL14, Wilms’ tumour 1-associating protein (WTAP) was found to act as another component of methyltransferase complex in mammals [8]. Both METTL3 and METTL14 possess methyltransferase activity and form stable heterodimer while WTAP does not although all of them share the same RNA binding motif. Loss of any of the components markedly decreases the m^6^A level as well as cell viability [7]. RNA-binding motif protein15 (RBM15) and its paralogue RBM15B were later reported to be parts of the complex facilitating its recruitment to specific sites on RNA molecules [9]. Recently few other components- Vir-like m^6^A methyltransferase associated protein (VIRMA)/KIAA1429, HAKAI and Zinc finger CCCH domain-containing protein13 (ZC3H13) have been found to interact with other components of the complex thus forming a large multi-component complex. In this complex VIRMA serves as a scaffolding protein holding WTAP, HAKAI and ZC3H13 together to create a binding site for METTL3 and METTL14 favoring their optimal catalytic function [10]. Besides these, an independent m^6^A writer has been discovered called METTL16, which catalyzes m^6^A modification in small nuclear RNA, U6 snRNA and other noncoding RNAs (lncRNAs) [11].

## Erasers

Fat mass and obesity-associated protein (FTO) was the first discovered m^6^A eraser which regulates cellular energy homeostasis and is associated with obesity. The role of FTO as m^6^A demethylase further revealed its association with gene expression regulation [12, 13]. ALKBH5 was identified as the second m^6^A eraser. Both FTO and ALKBH5 belong to Fe2^+^/α-ketoglutarate dependent dioxygenases enzyme family that are capable of recognizing adenine and cytosine methylation in single stranded DNA and RNA molecules. In mammalian cells a number of ALKB homologs have been identified which include ALKBH1–8, FTO and Tet 1–3, but other than FTO and ALKBH5 none of them showed substrate specificity towards mRNAs or lncRNAs [14, 15]. ALKBH3 has been found as a new m^6^A demethylase which only exhibits substrate specificity towards tRNAs [16, 17].

## Readers

While the methylation and demethylation is carried out by writers and erasers, it is the ‘readers’ who determines the functional outcomes of the modification. The YTH domain-containing family proteins1–3 (YTHDF1–3) were discovered as the first group of m^6^A readers [18]. YTH domain-containing protein1 (YTHDC1) was subsequently identified as a new m^6^A reader in a study to identify its mode of RNA-binding [19]. The fifth member of the family, YTHDC2 is an RNA helicase which like other family members contains YTH domain, but the feature that distinguishes it from others is the helicase domains that also contribute to RNA binding [20]. m^6^A-mediated conformational changes called ‘structural switch’ also contribute to exposure of RNA-binding motifs (RBMs) in proteins that are otherwise buried in their native structures giving rise to new group of m^6^A readers. Alterations in the structures of mRNA and lncRNA by m^6^A modification were found to facilitate the binding of heterogeneous nuclear ribonucleoprotein C (hnRNPC) which by recognizing m^6^A facilitates pre-mRNA processing [21]. Another member of this m^6^A reader family, hnRNPA2B1 plays vital roles in RNA splicing and microRNA (miRNA) processing [22]. The 5′-cap of an mRNA is typically required for protein translation. Eukaryotic initiation factor 3 (eIF3) was identified as an m^6^A reader, capable of initiating protein translation in a cap-independent manner in the presence of a single 5′-UTR m^6^A [23]. A distinct class of RNA-binding proteins named insulin-like growth factor-2 mRNA binding proteins 1, 2, and 3 (IGF2BP1–3) were identified as m^6^A readers by Huang et al. These proteins together with Hu antigen R (HuR) can recognize the m^6^A modifications in the transcripts, stabilizing them by protecting from degradation [24].

The subcellular localization of the writers, erasers and readers play crucial roles in determining the fate of m^6^A-modified transcripts and their biological roles (Table 1). The m^6^A readers and erasers are co-localized in the nuclear speckles, the primary site for mRNA processing and storage where they exert their methylation-demethylation effects on the target transcripts [25]. The reader proteins have both nuclear and cytoplasmic distribution indicating their functional diversity. The YTH domain containing proteins YTHDF1–3 and YTHDC2 are localized mainly in the cytoplasm while YTHDC1 is found in the nucleus. YTHDF1 recognizes the m^6^A modification around the stop codon and facilitates mRNA translation. YTHDF2 recognizes the mRNAs which are not destined for translation and accelerates their degradation. YTHDC1 regulates the splicing events by promoting splicing factor SRSF3 or inhibiting SRSF10. It can also interact with the nuclear RNA export factor 1 (NXF1) to facilitate export for mRNA out of the nuclei. YTHDC2 and YTHDF3 can enhance mRNA translation or facilitate mRNA degradation in a context-dependent manner. hnRNPs are localized in the nucleus where hnRNPC can bind and control the processing of the nascent RNA transcripts. hnRNPA2B1 by direct recognition of m^6^A participates in mRNA splicing and miRNA maturation [16, 25–27]. The IGF2BPs (IGF2BP1–3) upon colocalization with HuR in the P-bodies and upon translocation to the stress granules during heat shock, protect mRNA transcripts from degradation. They are also reported to participate in mRNA translation by regulating alternative splicing [28].

**Table 1:** Functions and subcellular localizations of m^6^A modification regulators (Writers, Erasers and Readers)

Regulators	Effectors	Cellular localization	Role
Writers	Methyltransferase-like 3 (METTL3)	Nucleus	Catalytic subunit of m^6^A methyltransferase complex
	Methyltransferase-like 14 (METTL14)	Nucleus	Catalytic subunit of m^6^A methyltransferase complex
	Methyltransferase-like 16 (METTL16)	Nucleus	Independent Methyltransferase
	Wilms’ tumour 1-associating protein (WTAP)	Nucleus	Enhancer of m^6^A methylation efficiency of methyltransferase complex
	RNA binding motifs protein 15 (RBM15)	Nucleus	Facilitates recruitment of methyltransferase complex on target RNA
	RNA binding motifs protein 15B (RBM15B)	Nucleus	Facilitates recruitment of methyltransferase complex on target RNA
	Vir-like m^6^A methyltransferase associated (VIRMA)/KIAA1429	Nucleus	Scaffolding protein for WTAP, HAKAI and ZC3H13 to facilitate binding with catalytic subunits
	HAKAI	Nucleus	Facilitates methyltransferase complex RNA binding
	Zinc finger CCCH domain-containing protein 13 (ZC3H13)	Nucleus	Facilitates methyltransferase complex RNA binding
Erasers	Fat mass and obesity-associated protein (FTO)	Nucleus	m^6^A demethylase of mRNAs and lncRNAs or other noncoding RNAs
	AlkB homologue5 (ALKBH5)	Nucleus	m^6^A demethylase of mRNAs and lncRNAs or other noncoding RNAs
	AlkB homologue3 (ALKBH3)	Cytoplasm	tRNA-specific m^6^A demethylase
Readers	YTH domain family proteins 1–3 (YTHDF1–3)	Cytoplasm	Facilitates mRNA translation (YTHDF1, YTHDF3) mRNAs degradation (YTHDF2, YTHDF3)
	YTH domain-containing protein 1–2 (YTHDC1–2)	Nucleus (YTHDC1) Cytoplasm (YTHDC2)	Regulates the splicing (YTHDC1) Context-dependent mRNA translation or degradation (YTHDC2)
	Eukaryotic initiation factor 3 (eIF3)	Cytoplasm	Cap-independent mRNA translation
	Insulin-like growth factor 2 mRNA-binding protein 1–3 (IGF2BP1–3)	Cytoplasm	Protect mRNA transcripts from degradation in stress condition mRNA alternative splicing
	Heterogeneous ribonucleoprotein C (hnRNPC)	Nucleus	Binds and controls processing of nascent RNA
	Heterogeneous ribonucleoprotein A2B1 (hnRNPA2B1)	Nucleus	mRNA splicing and miRNA maturation

## Oncogenic signaling pathways regulated/modulated by m^6^A methylation

**Fig. 2:**
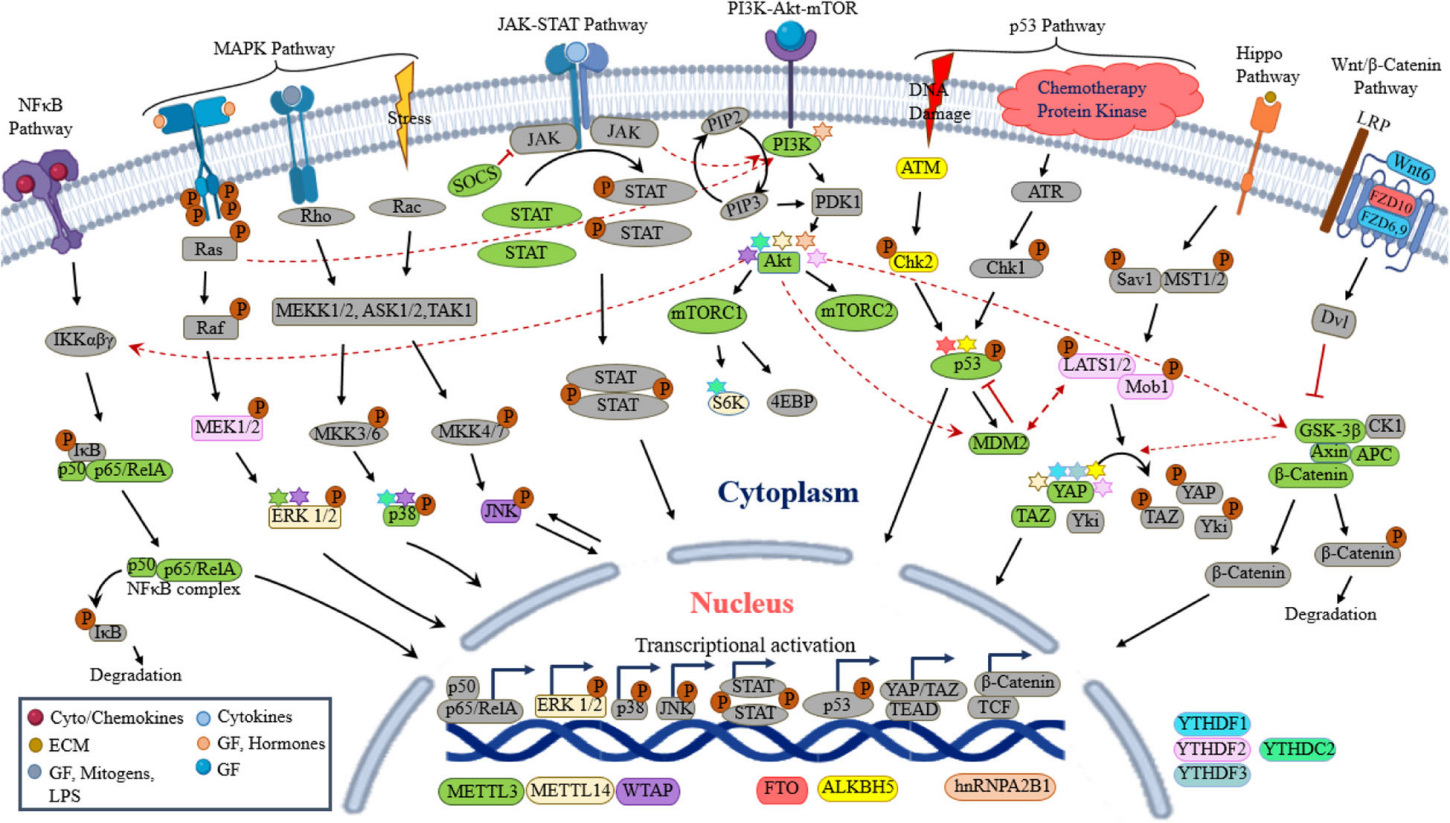
Molecular targets of m^6^A regulators in various signaling pathways. Individual members of m^6^A ‘Writers’, ‘Erasers’ and ‘Readers’ target specific RNA transcripts of cell signaling molecules causing activation/inactivation of various intracellular signaling pathways that play important roles in cancer. Targets of specific m^6^A regulators are indicated by the colors corresponding to the regulators. Molecules targeted by more than  one regulators are indicated by the ‘stars’ colored according to the regulatory proteins involved. GF: Growth Factor, ECM: Extracellular Matrix, LPS: Lipopolysaccharides

The process of carcinogenesis involves aberrant expressions of key signaling molecules that are tightly regulated by genes encoding them. Given the role of m^6^A modification in gene expression regulation it is likely to speculate that m^6^A may contribute to carcinogenesis via up or downregulation of vital components of cellular signaling. Here we compiled the crucial signaling molecules in key signaling pathways that are aberrantly expressed/activated/inactivated in different cancers due to m^6^A modification and the regulators that act upon them (Fig. 2, Table 2).

**Table 2:** Oncogenic signaling pathways modulated by m^6^A modification regulators

Signaling pathway	Cancer type	m^**6**^A regulator(s)	Up/downstream target(s)	Functional outcome/correlation	Reference
**Wnt/β-catenin**	Hepatoblastoma (HB)	↑ METTL3	↓ miR-186 (upstream of METTL3)	Increased proliferation, vascular invasion, metastasis, tumor recurrence	[29]
	Hepatoblastoma (HB)	↑ METTL3	↑ CTNNB1	Increased tumor growth	[30]
	Epithelial ovarian carcinoma (EOC)	↓ FTO ↓ ALKBH5 ↑ IGF2BP2	↑ FZD10	Resistance to PARP inhibitors (PARPi)	[31]
	Osteosarcoma (OS)	↑ METTL3	↑ Lymphoid enhancer-binding factor 1 (LEF1)	Increased tumorigenesis	[32]
	Pancreatic cancer (PC)	↓ ALKBH5	↓Wnt inhibitory factor 1 (WIF-1)	Increased cancer cell proliferation, migration, and invasion Enhanced chemoresistance	[33]
	Colorectal cancer (CRC)	↑ YTHDF1	↑ FZD9 and WNT6	Enhanced tumorigenicity and cancer stem cell formation	[34]
	Gastric cancer (GC)	↑ YTHDF1	↑ FZD7	Aggressive gastric tumor development	[35]
	Gastric cancer (GC)	↓ METTL3 ↓ METTL14 ↑ FTO	↑ β-Catenin ↑ Axin	Increased proliferation and invasiveness	[36]
**PI3K/Akt/mTOR**	Endometrial cancer	↓ METTL3 ↓ METTL14 (R298P Mut)	↓ PHLPP2 ↑ PRR5, PRR5L and mTOR	Increase proliferation and tumorigenicity	[37]
	Endometrial cancer	↑ FTO	Unspecified	Increased proliferation and invasiveness	[38]
	Gastric cancer (GC)	↓ METTL14 ↑ FTO	Unspecified	Increased proliferation and invasiveness	[36]
	Ovarian cancer	↑ METTL3	↑ miR-126-5p	Increased ovarian cancer cell proliferation, migration and invasion Decreasing apoptosis	[39]
	GI Cancers (Colorectal, gastric, esophageal, pancreatic, liver)	↓ METTL14 ↑ FTO	↑ PTEN, Akt1, PIK3CA and mTOR	Poor overall patient survival	[40]
	Nasopharyngeal carcinoma (NPC)	↑ YTHDC2	↑ Insulin-like growth factor 1 receptor (IGF1R)	Increased radiation resistance	[41]
	Renal cell carcinoma (RCC)	↓ METTL3	Unspecified	Cancer progression, decreased overall survival	[42]
	Cervical cancer	↑ hnRNPA2B1	Unspecified	Increased proliferation, migration and invasiveness	[43]
	Breast cancer	↑ FTO	Unspecified	Increased metabolic activity	[44]
	High-grade serous ovarian carcinoma (HGSOC)	↑ WTAP	Unspecified	Increased cell proliferation and migration Decreased apoptosis Poor overall patient survival	[45]
	Pancreatic cancer (PC)	↑ YTHDF2	↑ GSK3β	Increased cell proliferation	[46]
**JAK-STAT**	Hepatocellular carcinoma (HCC)	↑ METTL3 ↑ YTHDF2	↓ SOCS2	Increased HCC progression, poor overall and disease-free survival	[47]
	Colorectal cancer (CRC)	↑ METTL3	↓ SOCS2 ↑ LGR5	Increased stemness and chemoresistance	[48]
	Gastric cancer (GC)	↑ METTL3	↓ SOCS2	Increased cancer cell proliferation	[49]
**MAPK**					
ERK1/2	Renal cell carcinoma (RCC)	↓ METTL14 ↑ FTO ↓ RBM15B	↑ P2RX6	Increased RCC migration and invasion	[50]
p38/ERK	Colorectal cancer (CRC)	↓ METTL3	Unspecified	Increased tumor size, tumor advancement and metastasis	[51]
Raf/MEK/ERK	Colorectal cancer (CRC)	↑ METTL3	Pri-miR-1246 (↑ miR-1246)	Increased invasiveness and metastasis	[52]
Ras/Raf/ERK	Hepatocellular carcinoma (HCC)	↑ METTL3	↓ RDM1	Increased tumor progression	[53]
	Gastric cancer (GC)	↑ METTL3	↓ BATF2	Increased gastric carcinogenesis	[54]
p38 MAPK	Colorectal cancer (CRC)	↓ YTHDC2	Unspecified	Decreased apoptosis	[55]
	High-grade serous ovarian carcinoma (HGSOC)	↑ WTAP	Unspecified	Increased cell proliferation and migration Decreased apoptosis Poor overall patient survival	[45]
	Hepatocellular carcinoma (HCC)	↓ YTHDF2	↓ EGFR	Increased HCC cell proliferation	[56]
**p53 pathway**	Pancreatic cancer (PC)	↓ ALKBH5	↓ PER1	Increased cell proliferation and invasiveness	[57]
	Clear cell renal cell carcinoma (ccRCC)	↑ METTL3 ↑ METTL14	Unspecified	Poor disease prognosis and overall survival	[58]
	Esophageal carcinoma (ESCA)	↑ HNRNPC	Unspecified	Activation of cell cycle progression	[59]
	Colorectal cancer (CRC)	↑ METTL3	↑ mut-p53 mRNA	Increased chemoresistance	[60]
	Non-small cell lung cancer (NSCLC)	↑ FTO	↑ USP7	Increased cancer cell proliferation	[61]
	Hepatocellular carcinoma (HCC)	↑ METTL3	↓ RDM1	Increased tumor progression	[53]
**Hippo pathway**	Non-small cell lung cancer (NSCLC)	↑ METTL3 ↑ YTHDF1 ↑ YTHDF3	↑ YAP1 ↑ MALAT1 (lncRNA)	Increased invasiveness and chemoresistance	[62]
	Non-small cell lung cancer (NSCLC)	↑ ALKBH5	↓ YAP1	Decreased tumor growth, invasiveness, metastasis and EMT	[63]
	Lung adenocarcinoma (LUAD)	↑ METTL3	↑ TAZ	Increased cell growth, survival and invasiveness	[64]
	Colorectal cancer (CRC)	↓ METTL14	Pri-miR-375 (↓ miR-375)	Tumor progression Poor overall patient survival	[65]
	Pancreatic cancer (PC)	↑ YTHDF2	↑ Mob1	Suppressed migration, invasiveness and EMT	[46]
**NFκB pathway**	Malignant transformed hepatocyte stem-like cells	↑ METTL3	CUDR (upstream) SUV39H1 (downstream)	Malignant transformation of hepatocyte stem-like cells	[66]
	MCF7/ADR HepG2/ADR	↑ METTL3	↑ ERRγ	Chemoresistance	[67]
	Ovarian cancer	↑ ALKBH5	↑ NANOG	Carcinogenesis, increased cancer stem cells	[68]
	Bladder cancer (BCa)	↑ METTL3	↑ MYC	Increased cancer cell proliferation, migration and decreased apoptosis	[69]
**Hedgehog pathway**	Prostate cancer	↑ METTL3	↑ GLI1	Increased cancer cell survival, proliferation and invasiveness	[70]
**Notch pathway**	Glioma	↑ METTL3	↑ DLL1, DLL3, JAG2, NOTCH1–3 and HES1	Increased tumorigenesis	[71]
**Snail pathway**	Liver cancer	↑ METTL3 ↑ YTHDF1	↑ Snail	Increased tumor progression	[72]
	Hepatocellular carcinoma (HCC)	↑ METTL3	↑ SUMO1 (upstream) ↑ Snail (downstream)	HCC progression	[73]

## Wnt/β-catenin pathway

The Wnt/β-catenin pathway plays vital roles in developmental processes as well as maintaining adult tissue homeostasis. The key component of this signaling pathway is a glycoprotein Wnt which resides on the surface of the effector cell or is released from it. Wnt binds to its receptor complex on the target cell surface containing Frizzled (Fz) protein and low-density lipoprotein (LDL) receptor-related protein (LRP)5/6 [74]. Inside the cell there are several proteins that participate in transduction of the Wnt mediated signal which include- Dishevelled (Dvl), glycogen synthase kinase-3 (GSK-3), Axin, Adenomatous Polyposis Coli (APC) and β-catenin [75]. In the absence of ligand binding, cytoplasmic β-catenin is constantly degraded by the destruction complex containing Axin, APC, GSK-3 and the serine-threonine kinase CK1. When the Wnt binds to the receptor complex of Fz and LRP5/6, Dvl is recruited which phosphorylates LRP and recruits the Axin complex to the phosphorylated tail of LRP. This disrupts the Axin-scaffolded destruction complex inhibiting the phosphorylation of β-catenin and proteasomal degradation. As a result, the β-catenin is accumulated in the cytoplasm and translocated to the nucleus where it interacts with and activates TCF/Lef1 transcription complex initiating Wnt-activated gene expression [74, 76, 77].

Altered m^6^A modification and its regulatory protein expression have been observed in different cancer types modulating the Wnt/β-catenin signaling pathway. METTL3 has been found to highly express in hepatoblastoma (HB) which is linked to advanced clinical features like- vascular invasion, distant metastasis or HB recurrence [29, 30]. In molecular level miR-186 have been found to directly target the METTL3 decreasing its expression by directly binding to the 3′-UTR of METTL3 mRNA. In HB tumors the expression of miR-186 was significantly downregulated causing high METTL3 protein expression. This miR-186/METTL3 axis has been found to regulate the Wnt/β-catenin signaling pathway to promote cell proliferation, migration and invasion in HB. With high METTL3 expression and low miR-186 expression, the Wnt/β-catenin pathway-associated proteins such as- β-catenin, APC, cyclin D1 and c-myc were also found overexpressed [29]. METTL3 also regulates the Wnt/β-catenin pathway in HB by increasing the m^6^A level of important genes in the pathway such as CTNNB1 (β-catenin), CCND1 (cyclin D1), NKD1 (NKD inhibitor of Wnt signaling pathway 1) etc. In HB a significant positive correlation has been found between METTL3 and CTNNB1 gene that encodes for β-catenin. Knockdown of METTL3 downregulated the expression of CTNNB1 expression and stability by decreasing m^6^A methylation of CTNNB1 transcripts indicating CTNNB1 as a direct downstream target of METTL3 in HB cells [30].

m^6^A mRNA methylation has also been reported to play oncogenic roles in PARP inhibitor (PARPi) resistant BRCA-mutated epithelial ovarian cancers (EOC). It was found that FZD10, a receptor and a modulator of Wnt/β-catenin signaling was upregulated due to increased m^6^A methylation at the 3’UTR region of the FZD10 mRNA. Although the levels of METTL3 and METTL14 did not increase significantly in these cells, the demethylases FTO and ALKBH5 were downregulated which was sufficient to increase m^6^A level in FZD10 mRNA. Also, the m^6^A reader IGF2BP2 which plays a role to stabilize m^6^A-containing mRNAs was upregulated in these cells. Increased m^6^A in FZD10 mRNA thus increased the β-catenin level in the nuclei thereby upregulating its target genes FOSL1 and CCND1 [31].

In human osteosarcoma (OS), METTL3 and m^6^A methylation were found upregulated which activated the oncogenic Wnt/β-catenin signaling. Lymphoid enhancer-binding factor1 (LEF1), an important component of Wnt signaling was the target of METTL3. With METTL3 upregulation, the m^6^A methylation of LEF1 mRNA was increased which also increased its stability causing Wnt/β-catenin pathway activation [32]. Low ALKBH5 expression was correlated with increased pancreatic adenocarcinoma progression and chemoresistance due to Wnt/β-catenin pathway activation. ALKBH5 overexpression blocked this pathway by m^6^A demethylation-mediated upregulation of Wnt inhibitory factor-1 (WIF-1) which in pancreatic cancer was overcome by ALKBH5 downregulation [33]. The m^6^A reader YTHDF1 overexpression was found to positively correlate with the colorectal cancer (CRC) progression via Wnt/β-catenin pathway activation. In CRC cells YTHDF1 recognition of the m^6^A-modified transcripts of Wnt signaling components FZD9 and WNT6 enhanced their translation. Abnormally increased FZD9 and WNT6 proteins then activated the Wnt/β-catenin pathway to enhance tumorigenicity and CRC stem cell formation [34]. YTHDF1 overexpression has also been correlated with gastric cancer progression via m^6^A- mediated FZD7 mRNA translation and Wnt/β-catenin hyper-activation [35].

## PI3K-Akt-mTOR pathway

The PI3K-Akt-mTOR pathway is an interconnected cellular signaling pathway that is crucial in many cellular processes including growth and survival, nutrient uptake, anabolic reactions as well as in pathological conditions, particularly cancers [78, 79]. Phosphatidylinositide 3-kinases (PI3Ks), Akt/protein kinase B (PKB) and mammalian target of rapamycin (mTOR) are the core components of this pathway. PI3Ks are a family of intracellular lipid kinases that phosphorylate the 3′-hydroxyl group of the inositol ring of phosphatidylinositides (PIs) [80]. Upon activation by growth factor receptors, the PI3Ks are recruited to the cell membrane that catalyzes the conversion of phosphatidylinositol-(4,5)-bisphosphate (PI-4,5-P2) to phosphatidylinositol-(3,4,5)-trisphosphate (PI3,4,5-P3). PI3,4,5-P3 or PIP3 acts as a second messenger to initiate the signaling by activating and recruiting the downstream proteins such as PI3K-dependent kinase-1 (PDK1) and Akt. Upon activation Akt can activate/inactivate several other downstream proteins including glycogen synthase kinase 3 (GSK3), IκB kinase (IKK), tuberous sclerosis 2 (TSC2), Bad, caspase 9 and PRAS40 (AKT1S1) etc. By this way Akt can exert a wide range of biological functions including promoting cell proliferation, survival, differentiation, apoptosis, angiogenesis and metabolism [79]. Akt acts as a positive regulator of mammalian target of rapamycin (mTOR) upon activation of vascular endothelial growth factor receptors (VEGFRs). mTOR is a serine/threonine kinase which acts as a catalytic subunit of two distinct protein complexes: mTOR complex1 (mTORC1) and complex2 (mTORC2). Activation of mTORC1 phosphorylates a number of downstream proteins such as S6K1, 4EBP, RPS6K, ULK1 etc. and regulates vital cellular functions like lipid and nucleic acid synthesis, protein synthesis and autophagy thereby regulating cell growth. mTORC2 on the other hand acts on proteins like Akt, PKA, PKC, SGK etc. and regulates cellular functions such as cytoskeletal organization, cell migration, lipid metabolism controlling overall cell survival and proliferation [79–81].

Altered expression of the components of PI3K-Akt-mTOR pathway has been reported with alterations in the m^6^A mRNA methylation or its regulators in different cancer types. In endometrial cancer, a decrease in the m^6^A mRNA methylation in the mRNA transcripts of the important Akt-mTOR pathway components PHLPP2 (a phosphatase causing Akt dephosphorylation and inactivation) and mTORC2 (which phosphorylates and activates Akt) due to a hotspot R298P mutation in METTL14 and simultaneous downregulation of METTL3 contributed to increased proliferation and tumorigenicity. Decrease in m^6^A methylation of PHLPP2 mRNA decreased its expression, while decreased m^6^A methylation in mRNA transcripts of the components of mTORC2 complex (PRR5, PRR5L and mTOR) increased their stability and expression. Increased Akt activation with decreased m^6^A methylation of the target transcripts due to mutation in METTL14 or METTL3 downregulation contributed to endometrial cancer progression [37]. Similarly, decreased m^6^A methylation due to decreased METTL3 and increased FTO was also associated with increased tumorignesis in gastric cancer (GC). With METTL3 knockdown the PI3K-Akt pathway was activated as evidenced by Akt and S6 phosphorylation. METTL3 knockdown also activated Wnt/β-catenin pathway as indicated by β-catenin and axin upregulation and GSK-3β phosphorylation. The opposite effect was observed with FTO downregulation. These results indicated a regulator-mediated effect of m^6^A methylation changes in the target transcripts to be the cause of increased proliferation and invasiveness of gastric cancer cells [36]. Conversely, increased METTL3 expression has been shown to promote ovarian cancer by m^6^A modification mediated maturation and upregulation of miR-126-5p. In cancer cells miR-126-5p inhibited PTEN expression causing PI3K/Akt/mTOR pathway activation [39].

Using bioinformatics databases and different gastrointestinal (GI) cancer cell lines Zhao et al. showed that m^6^A methylation also regulates PI3K-Akt-mTOR pathway in GI cancers by affecting the stability of proteins acting in this pathway. In this study they showed that, m^6^A methylation in the PTEN, Akt1, PIK3CA and mTOR transcripts decreased their stability and translation. Silencing the METTL3 expression or inhibiting the methyltransferase activity of METTL3-METTL14 writer complex by S-adenosylhomocysteine (SAH) increased their stability and expression but inhibiting the demethylase activity of FTO by meclofenamic acid (MA) had an opposite effect. In addition, the intracellular kinase activity of Akt1, mTOR, GSK3β and p38 was inhibited upon SAH treatment but promoted by MA indicting a modulatory effect of m^6^A modification on PI3K-Akt-mTOR signaling pathway [40]. In nasopharyngeal carcinoma (NPC), an increased expression of m^6^A reader YTHDC2 was correlated with the activation of PI3K-Akt/S6 signaling conferring radioresistance to the cancer cells. YTHDC2 was found upregulated in radioresistant NPC cells compared to their radiosensitive counterparts. YTHDC2 activated PI3K-Akt/S6 signaling by increasing the translation of insulin-like growth factor-1 receptor (IGF1R) mRNA [41]. Inverse correlation of METTL3 expression and renal cell carcinoma (RCC) progression and development was reported in another study. In RCC cells, PI3K-AKT-mTOR pathway was activated with decreased METTL3 expression as indicated by increased phosphorylation of the components of this pathway such as p-PI3K, p-AKT, p-mTOR, and p-P70. Upon METTL3 overexpression the levels of these phosphoproteins decreased indicating a suppressive effect of METTL3 on PI3K-AKT-mTOR pathway [42].

The m^6^A reader hnRNPA2B1 has been reported to be upregulated in cervical cancer which activated the PI3K-Akt pathway promoting cancer cell proliferation, invasion and migration [43]. The m^6^A demethylase FTO expression was found to be induced by estrogen in endometrial cancer via activation of PI3K-Akt and MAPK pathway where FTO was the downstream target of PI3K-Akt and MAPK [38]. FTO also activated the PI3K-Akt pathway in breast cancer cells which promoted the glycolysis and lactic acid production [44]. Although m^6^A methylation changes in any of the mRNA transcripts in the PI3K-Akt pathway was not tested in these studies, it is possible that FTO or hnRNPA2B1 may be involved in regulating their target gene expressions via changing or recognizing their methylation status.

## JAK-STAT pathway

The Janus kinase (JAK)-signal transducer and activator of transcription (JAK-STAT) signaling pathway is associated with cellular proliferation, migration, differentiation and apoptosis aberrant expression of which is linked to different types of cancers [82]. JAK signaling is transduced through the activation of STAT and its target genes which is considered as the canonical mode of signal transduction. However, a non-canonical mode of JAK-STAT signaling was also discovered. In mammalian cells there are four JAK kinase proteins (JAK1–3 and TYK2) which act upon seven STAT proteins (STAT1–4, 5A-B and 6). The cascade of JAK-STAT signal transduction is initiated with the binding of a peptide ligand (such as growth factor or cytokine) to its transmembrane receptor [83, 84]. JAK is associated with the cytoplasmic domains of the receptors in its inactive form. Ligand binding to the receptor causes tyrosine phosphorylation of the cytoplasmic domains of the receptors and causes receptor multimerization which brings two units of JAK kinase in close proximity to each other allowing a transphosphorylation to occur. JAK activation by the receptors in turn causes phosphorylation of the tyrosine residues in the cytoplasmic domains of the receptors which act as docking sites for cytoplasmic STAT proteins. Binding of STAT proteins to the receptor bring them to the JAK kinases which then phosphorylate the tyrosine residues in the C-terminal of STAT proteins and activate them. Upon phosphorylation STATs form homo- or heterodimers and dissociate from the receptor complex translocating to the nucleus. In the nucleus the STAT dimers bind with the promoter regions of their target genes and activate/repress their transcription [83, 85]. The activity of JAK-STAT pathway is negatively regulated by three different class of proteins: suppressors of cytokine signaling (SOCS), protein inhibitors of activated stats (PIAS) and protein tyrosine phosphatases (PTPs). STAT proteins upon activation and translocation in the nucleus activates the transcription of SOCS genes and the resulting SOCS proteins then bind to the JAKs and their associated receptors thus inactivating them. The PIAS proteins on the other hand bind to the STAT dimers and inhibit their binding to the target genes intended for transcription. The PTPs interact with the phosphorylated receptors in the cell membrane or the attached phosphorylated JAKs leading to their dephosphorylation and inactivation [82, 86].

Expression of the components of this pathway may be regulated in the transcriptional level by m^6^A leading to abnormal signaling in cancer progression. In hepatocellular carcinoma (HCC), METTL3 has been found to upregulate in cancerous cells compared to normal hepatocytes. Knockdown of METTL3 in HCC cells decreased their proliferative and metastatic capability as well as tumorigenicity in nude mice. On the other hand, METTL3 overexpression promoted these effects. SOCS2 has been identified as the target for METTL3-mediated m^6^A modification which is recognized by YTHDF2 leading to its degradation. Conversely, SOCS2 overexpression due to METTL3 knockdown increased the expression of phosphorylated STAT5 indicating a regulatory role of m^6^A on the JAK-STAT pathway in HCC progression [47]. Elevated METTL3 level with subsequent downregulation of SOCS2 was also observed in colorectal cancer (CRC). Increased m^6^A in SOCS2 mRNA decreased its stability contributing to low SOCS2 protein expression in cancerous cells which abolished its inhibitory effect on the leucine-rich repeat-containing G protein-coupled receptor5 (LGR5) expression. LGR5 is responsible for stemness and chemoresistance in colon cancer cells. Thus, increased post-transcriptional m^6^A modification of SOCS2 promoted cancer cell proliferation via LGR5 induction in CRC [48]. Increased METTL3 expression and decreased SOCS2 protein level was also reported to increase cellular proliferation in gastric cancer cells although no STAT activity could be established in this study [49].

m^6^A RNA modification may also contribute to T cell-mediated tumor immunity as METTL3 has been reported to regulate T cell homeostasis and differentiation in knockout animal models. METTL3-mediated m^6^A modification was found to control the balance between IL-7- mediated JAK-STAT signaling and TCR-mediated ERK-AKT signaling, two important cellular signaling pathways in T cells regulating their homeostasis. Thus m^6^A accelerates SOCS1, SOCS3 and CISH mRNA degradation activating the IL-7-mediated JAK signaling and the downstream STAT5 activation to re-program the naïve T cell for differentiation and proliferation [87].

## MAPK pathway

The mitogen-activated protein kinase (MAPK) family proteins are the family of kinases that transmit extracellular signals to the intracellular components which control fundamental cellular processes like growth and differentiation, proliferation, motility, stress response, survival and apoptosis. MAPK pathway converges signals from a wide range of stimuli including stress and DNA damage responses, growth factors, extracellular matrices and responses from cell-cell interaction [88]. Upon receiving signals, the pathway activates the MAPKs in a three-tier kinase activity in which the receptor stimulation first phosphorylates the MAPK kinase kinase (MAPKKK) which in turn phosphorylates MAPK kinase (MAPKK) and MAPK is then phosphorylated and activated by MAPKK. Activation of MAPK pathway act upon diverse cellular components and initiate a series of signaling events. Depending on the type of kinase in the MAPK layer, there are at least four cascades of MAPK pathways: extracellular signal-regulated kinase (ERK)1/2, c-Jun N-terminal kinase (JNK)1/2/3, p38 signaling isoforms (p38 α/β/γ/δ) and ERK5 [89, 90]. The ERK1/2 pathway is the first MAPK cascade discovered and the most well studied of all MAPK pathways. The signaling in ERK pathway is transduced mainly by the Ras-Raf-MEK module where the extracellular signal induce the phosphorylation of small GTPase Ras proteins. Ras in turn recruit and phosphorylates Raf which on its downstream phosphorylates MEK 1 and 2. MEK1/2 then phosphorylates the MAPKs- ERK 1 and 2 and the signal continues by phosphorylation of hundreds of substrates to their downstream in the cytoplasm, nucleus and various other organelles [90, 91]. The JNK cascade was identified as the activator of the transcription factor c-Jun (hence named as JNK) in response to intra and extracellular stresses. The JNK cascade initiates with the activation of small GTPases Rho, Rac and CDC42 which then phosphorylate the MAPKKKs MEKK1/2, MLK1/2, TAK1, ASK1/2, TAO1 etc. Activation of MAPKKKs further phosphorylates the kinases MKK4 and MKK7. Activated MKKs in turn activate the MAPKs JNK1–3 which shuttle between cytoplasm and nucleus and act on their downstream targets such as ATF2, HSF1, STAT3, p53 etc. [91, 92]. Another stress-activated MAPK cascade is p38 pathway which is activated by the same small GTPases and MAPKKK components of JNK cascade but differs from that by the MAPKK components (MKK3 and MKK6) as JNK activators [89, 92]. Unlike other MAPK cascades ERK5 is less studied and hence less understood.

Dysregulated MAPK signaling has been implicated in numerous cancer types. However, the contribution of m^6^A RNA methylation on regulating this pathway in cancer has started to emerge only in recent days. Decreased METTL14 and increased carcinogenesis in renal cell carcinoma (RCC) has been reported in a recent study. In this study it is shown that, increased m^6^A modification is negatively correlated with cancer cell migration and invasion through alternative splicing and downregulation of P2RX6, a G-protein coupled Ca^2+^ ion channel and a preferred receptor for ATP binding. Overexpression of this receptor increases Ca^2+^ influx in the RCC cells which activates the ERK1/2 pathway. ERK1/2 activation increases the transcription of MMP9 which triggers the invasion and migration of RCC cells. METTL14 downregulation in cancer cells decreases the m^6^A levels in P2RX6 mRNA, thereby increasing its expression which leads to ERK1/2 MAPK pathway activation promoting RCC cell migration and invasion [50].

Decreased METTL3 was found correlated with increased tumor size, advancement and metastasis in colorectal cancer (CRC) where METTL3 downregulation activated the MAPK pathway-associated proteins- particularly p38 and ERK resulting in CRC cell proliferation, migration and invasiveness [51]. An opposite effect of METTL3 in CRC was also reported. In this case increased m^6^A RNA modification was observed in cancerous and surrounding normal tissues due to upregulation of METTL3 which was otherwise low in the normal tissues. METTL3 increased the m^6^A modification in the pri-miR-1246 promoting its maturation which suppressed the transcription of its downstream target SPRED2 that activates RAF/MEK/ERK pathway. Suppression of SPRED2 inhibits the phosphorylation of RAF/MEK/ERK proteins and abolishes its suppressive effect on migration and invasive capacity of cancer cells. METTL3-mediated m^6^A modification and upregulation of miR-1246 thus negatively regulate SPRED2 and inactivate MAPK pathway to promote cancer metastasis [52]. METTL3 overexpression contributed to the gastric cancer progression by m^6^A-mediated downregulation of basic leucine zipper ATF-like transcription factor2 (BATF2). BATF2 acts as a tumor suppressor by enhancing p53 protein stability thereby inhibiting ERK signaling. METTL3-mediated downregulation of BATF2 reversed this effect [54].

Low YTHDC2 expression was observed in cancerous tissues compared to normal tissues in CRC patients. Bioinformatic analysis showed a positive correlation of YTHDC2 with MAPK pathway and downstream components of intrinsic and extrinsic apoptotic pathways. YTHDC2 activated the p38 MAPK which promoted transcription of a number of genes causing upregulation of exogenous death receptor pathway associated proteins caspase 8, Fas and FasL and the endogenous mitochondrial apoptotic pathway proteins Bax, caspase 9 and cytochrome C [55]. WTAP has been found highly expressed in high-grade serous ovarian carcinoma (HGSOC). Knockdown of WTAP in ovarian cancer cells decreased expression of several MAPK proteins such as p-ERK, ERK, p-JNK, JNK, p38 and p-p38 as well as AKT pathway proteins (p-AKT, AKT) indicating the association of these two pathways with WTAP in ovarian cancer [45]. In hepatocellular carcinoma (HCC), YTHDF2 was downregulated in response to hypoxia leading to HCC cell proliferation. Overexpression of YTHDF2 abolished the hypoxic stress induced MAPK pathway activation by decreasing the phosphorylation of ERK and MEK. YTHDF2 exerted its suppressive effect on MAPK pathway by directly targeting its upstream regulator EGFR recognizing and interacting with m^6^A on EGFR mRNA thereby promoting its degradation. Thus, YTHDF2 overexpression inhibited the MEK/ERK pathway which was activated by hypoxia and suppresses HCC growth and proliferation [56].

## p53 pathway

p53 is a well-known tumor suppressor which is also termed as the ‘guardian of the genome’. It acts as a cellular stress sensor which upon activation by cellular stresses like DNA damage, hypoxia, hyperproliferation, oxidative stress or lack of nutrients triggers feedback responses such as cell cycle arrest, senescence or apoptotic cell death [93]. In a normal cell at its basal state, p53 level is maintained in a steady state level by several negative regulators such as Mdm2, COP1, ARF-BP1 and Pirh2. The p53 regulators are all ubiquitin ligases which bind to p53 to make it a target for degradation by proteasomal pathway [94]. p53 network of proteins are activated by three independent pathways- a) by DNA damage, b) in response to aberrant growth signals and c) chemotherapeutic agents or protein kinase inhibitors. DNA damage by the ionizing radiation recruits the protein kinases called ataxia telangiectasia mutated (ATM) and Chk2 kinases. Activation of ATM is caused by the DNA double-strand break which in turn activates Chk2. Aberrant growth signals triggered by oncogenes like Ras or Myc causes activation of p14^ARF^ protein which activates the p53 network. p53 activation by chemotherapeutic agents or protein kinases are brought about by a group of kinases called ataxia telangiectasia related (ATR) proteins. Activation of these pathways stabilize the p53 protein by inhibiting its degradation resulting in high p53 level inside the cells. Increased p53 level thus induces the transcriptional activation of the downstream signaling proteins to carry out its functions [95].

Alterations in the m^6^A status in mRNA transcripts due to changes in m^6^A regulatory components have been reported to alter the expression of genes that are involved in p53 signaling pathway in various cancer types. Dominissini et al. showed that, knockdown of the METTL3 resulted in changes in more than 20 genes that are associated with p53 signaling pathway. These alterations in p53 pathway proteins which include Mdm2, Mdm4 and p21 are due to alternative mRNA splicing resulting from altered m^6^A status [18]. In acute myeloid leukemia (AML) patients, coexistence of mutations and/or copy number variations of the m^6^A regulatory genes and p53 mutations were observed which conferred a poor overall patient survival. However, it is not clear how or to what extent the m^6^A regulators are contributing to the incorporation of the mutation marks in p53 gene [96]. Copy number variations of m^6^A regulatory genes and alterations of TP53 status were also observed in clear cell renal cell carcinoma (ccRCC) patients which is associated with poor disease prognosis and overall survival. In these patients the METTL3 and METTL14 transcripts and protein expression were higher than their normal counterparts which are assumed to be significantly associated with TP53 alteration [58].

In an effort to identify and validate the prognostic signature of the m^6^A regulators in esophageal carcinoma (ESCA), Yang and colleagues observed several m^6^A regulatory proteins that were upregulated in ESCA. Among these, high expression phenotype of HNRNPC was associated with activation of cell cycle and p53 signaling pathway [59]. Low expression of m^6^A eraser ALKBH5 was found in a cohort of pancreatic cancer (PC) patients in which both the mRNA and protein levels of ALKBH5 were downregulated in the cancerous tissues compared to the noncancerous pancreatic tissues. Overexpression of ALKBH5 inhibited the proliferation and tumorigenic potential of the PC cells in both in vitro and in vivo studies. RNA-seq analysis revealed PER1 mRNA, as the most affected transcript which was downregulated upon ALKBH5 loss through elevated m^6^A and recognition by YTHDF2. Ectopic expression of PER1 in PC cells activated the p53 pathway in an ATM-dependent manner as evidenced by the upregulation of p-ATM, p-CHK2, p-CDC25C, p-p53, p21, and p-CDK1 as well as downregulation of Cyclin B1 indicating a positive regulation of p53 pathway by ALKBH5. Ectopic p53 expression in these cells on the other hand increased ALKBH5 level by direct interaction with its promoter and decreased overall m^6^A level in the transcripts creating a positive feedback loop. Thus, increased m^6^A in PC promoted tumor growth and invasiveness due to interruption of the ALKBH5-PER1-p53-ALKBH5 feedback loop [57].

Increased m^6^A modification due to METTL3 overexpression was also found to contribute to the mutant p53 expression and increased chemotherapeutic resistance in colon cancer cells. Elevated glycosphingolipid (particularly Gb3) in the glycosphingolipid-enriched microdomains (GEMs) of the cancer cell membrane due to increased glucosylceramide synthase (GCS) activated the cSrc/β-catenin signaling pathway which upregulated the METTL3 expression. METTL3 overexpression increases the m^6^A modification in the point mutated codon 273 of p53 pre-mRNA with preferential splicing towards mutant protein expression. Suppression of glycosphingolipid Gb3 production or METTL3 silencing suppressed the m^6^A modification in p53 pre-mRNA and activated the p53 pathway as evidenced by increased p-p53, p21, PUMA and Bax protein expression thereby resensitizing the cells to chemotherapeutic agents [60, 97].

FTO was found upregulated in human non-small cell lung cancer (NSCLC) cells increasing their rate of proliferation. FTO exerted the protumorigenic effects by decreasing the m^6^A level in the ubiquitin-specific protease-7 (USP7) mRNA thereby stabilizing and increasing USP7 protein expression that played an important role in p53 pathway-dependent tumor progression. Inhibition of USP7 promoted the degradation of E3-ubiquitin ligase Mdm2 inhibiting the ubiquitination and degradation of p53 causing tumor suppression. FTO stabilized the USP7 transcript and caused its upregulation thereby promoting the growth of NSCLC cells by inactivating p53 pathway [61].

In hepatocellular carcinoma (HCC), METTL3 has been shown to play a protumorigenic role by decreasing the level of RDM1, a key regulator of the DNA double-strand break repair and recombination, RNA processing and protein translation. RDM1 was found to bind with p53 protein prolonging its half-life and modulate the downstream protein p21, Cyclin A1 and 14–3-3σ thus acting as a tumor suppressor. RDM1 was also found to repress the Ras/Raf/ERK signaling pathway in the presence of wild-type p53. METTL3 induced the m^6^A modification to the RDM1 transcripts which decreased their stability and repressed RDM1 protein expression [53].

## Hippo pathway

The Hippo signaling pathway is one of the key pathways for controlling organ development or maintaining tissue homeostasis. Hippo pathway maintains it by promoting cell death or inhibiting cellular proliferation or differentiation deregulations of which are also implicated in pathological conditions like cancers [98]. The main components of this pathway are: mammalian Ste20-like kinases 1/2 (MST1/2), large tumor suppressor 1/2 (LATS1/2), Salvador homolog 1 (Sav1), MOB kinase activator 1A and 1B (MOBKL1A and 1B) collectively known as Mob1, Yes association protein (YAP) and TAZ (also known as WWTR1). MST1/2, LATS1/2 and Mob1 form a cascade of kinases which act on YAP and TAZ to phosphorylate and inactivate them [99]. In canonical Hippo signaling, activation of the pathway MST1 and 2 kinases are phosphorylated which further phosphorylate and activate LATS1/2. Activation of LATS1/2 is also carried out by Mob1 kinases. Mob1 is also phosphorylated by MST1/2 which enhances Mob1-LATS1/2 interaction. Sav1 has been shown to act as a scaffolding protein for the Hippo pathway components which can also bind and phosphorylate MST1/2 which in turn phosphorylate Sav1 [98, 99]. LATS1/2 can directly interact with and phosphorylate their downstream target YAP, TAZ and Yki. Phosphorylation of these proteins promotes their retention in the cytoplasm by interaction with the 14–3-3 proteins where they undergo proteasomal degradation. YAP, TAZ and Yki are the downstream effectors of the Hippo pathway, phosphorylation and degradation of which inhibits their nuclear translocation and termination of their activity as transcriptional coactivators [98, 100]. When the Hippo pathway is inactive, YAP, TAZ and Yki proteins translocate to the nucleus where they activate the transcriptional process of their downstream target genes, mainly the TEA-domain-containing (TEAD) family members TEAD1–4, the transcription of which are associated with cell proliferation and death. They also regulate the transcriptional activity of SMAD1/2/3, SMAD7, RUNX1/2, p73 and ErbB4 which are associated with transcription of a wide array of genes involved in cell proliferation, differentiation or developmental processes [98, 99, 101]. With the increasing evidence that YAP and TAZ are highly expressed in solid tumors, these molecules are emerging as crucial determinants of malignant transformations in human cancers [100, 102].

The main determinants of the outcomes of Hippo pathway YAP and TAZ expression have been found to be regulated by m^6^A modification in human tumors. Increased m^6^A modification due to increased METTL3 expression and YAP upregulation was observed in human NSCLC cells compared to normal lung cells. Elevated METTL3 level in cancer cells increases m^6^A modification in YAP mRNA transcripts which are recognized by m^6^A readers YTHDF1 and YTHDF3 that are also upregulated in NSCLC cells. This subsequently recruits the translation initiation factor eIF3b resulting in an increased YAP level. In addition to this, elevated METTL3 and YTHDF3 also increased the stability and expression of lncRNA MALAT1 which further upregulated the YAP expression via modulation of miR-1914-3p. These all together enhanced tumor growth, metastasis and impart drug resistance to NSCLC cells [62]. In NSCLC, ALKBH5 was found to decrease tumor growth, invasiveness and metastasis by decreasing m^6^A-mediated YAP expression and inactivation. The m^6^A reader YTHDF2 recognized the YAP mRNA recruiting argonaute RISC catalytic component 2 (AGO2) to facilitate YAP mRNA degradation. ALKBH5 further decreases the YAP activation by decreasing the miR-107 level in a HuR dependent manner. Thus, reducing m^6^A methylation of YAP by ALKBH5 contributes to decreased tumor growth and metastasis by YTHDF2-mediated YAP degradation and miR-107/HuR-mediated YAP inactivation [63].

Elevated METTL3 expression was also found to increase translation of oncoproteins- EGFR and Hippo pathway effector TAZ thereby increasing growth, survival and invasiveness of lung adenocarcinoma (LUAD) cells. METTL3 promoted the translation of target mRNAs by direct recruitment of eIF3 [64]. An opposite effect of m^6^A methylation in the transcripts was observed in colorectal cancer (CRC) due to YAP1-mediated Hippo pathway activation. In CRC a decreased METTL14 level was observed which resulted in a decreased m^6^A methylation in the pri-miR-375 inhibiting its maturation by DGCR8. Decreased miR-375 subsequently resulted in upregulation of oncogenes YAP1 and SP1 promoting the tumor progression. In contrast, overexpression of METTL14 inhibited CRC progression by miR-375-mediated downregulation of YAP1 and SP1 [65].

YTHDF2 was found upregulated in pancreatic cancer which promoted cancer cell proliferation via activation of Akt/GSK3β/Cyclin D1 pathway. However, YTHDF2 overexpression also suppressed the migration and invasive capability of pancreatic cancer cells, an effect termed ‘migration-proliferation dichotomy’. YTHDF2 suppressed the EMT by upregulation and phosphorylation of Mob1 which in turn phosphorylated and activated LATS1 and activated pLATS1 further phosphorylated and inactivated YAP [46].

## Other pathways

Apart from the pathways discussed above, several other cellular signaling pathways such as NFκB, TGFβ, Hedgehog signaling pathway, Notch signaling pathway etc. have also been reported to be either directly or indirectly modified by m^6^A methylation or its regulatory proteins leading to malignant transformations and cancer development.

NFκB is a regulator of the transcriptional activity of the genes that are involved in inflammation, cell proliferation, survival and malignant transformation. It is shown that, IL-6 in cooperation with lncRNA CUDR induced the malignant transformation of the human embryonic stem cells derived hepatocyte-like stem cells through activation of NFκB signaling by METTL3. Triggered by inflammatory cytokine IL-6, the lncRNA CUDR overexpression enhanced the expression of METTL3 and increased m^6^A mRNA methylation of the suppressor of variegation 39 homolog-1 (SUV39H1), a histone methyltransferase thereby increasing its expression. SUV39H1 in turn enhanced Histone3 mono-, di- and tri-methylation leading to the expression and phosphorylation of NFκB. Activated p-NFκB translocated to the nucleus and promoted expression and phosphorylation of STAT3. By interacting with the promoter regions of target miRNAs and lncRNAs, p-STAT3 caused the malignant transformation of the hepatocyte-like stem cells [66]. Cytokines released in the tumor microenvironment are also correlated with NFκB activation and tumor progression via changes in cellular m^6^A status. Ovarian cancer cells co-cultured with macrophages showed higher expression of m^6^A demethylase ALKBH5 and TLR4. ALKBH5 was also found upregulated in ovarian cancer tissues compared to normal ovarian tissues. It was found that the increased ALKBH5 level was due to TLR4-mediated activation of NFκB pathway. Increased ALKBH5 further targeted the NANOG transcript by increasing m^6^A level, therefore contributing to increased ovarian cancer stem cell generation and carcinogenesis [68]. Increased METTL3 and m^6^A-mediated upregulation of estrogen receptor related receptors γ (ERRγ) was shown to trigger chemoresistance in cancer cells. ERRγ interacted with NFκB/p65 to induce transcriptional activation of ABC transporter ABCB1 (thereby increasing expression of P-gp) which facilitated drug efflux [67]. METTL3 has also been found upregulated in bladder cancer (BCa) which promoted proliferation, migration and suppresses the apoptotic cancer cell death. This oncogenic effect of METTL3 is mediated by multiple levels of regulation on MYC oncogene, one of which is activation of NFκB pathway [69].

METTL3 also exhibited its regulatory effect on Hedgehog pathway promoting prostate cancer cell proliferation, survival and invasive capability. METTL3 exerted these effects by enhancing the expression of GLI1, one of the key Hedgehog signaling proteins which contributed to prostate cancer progression by upregulation of downstream targets c-Myc and cyclin D1 [70].

In glioma stem-like cells METTL3 was found upregulated which contributed to tumorigenesis via activation of Notch signaling. Several Notch signaling genes (such as notch ligands DLL1, DLL3 and JAG2, notch receptors NOTCH1, NOTCH2 and NOTCH3, and notch target HES1) were found to have enhanced m^6^A modification which resulted in their upregulation [71]. METTL3 upregulation has also been implicated in promoting migration and invasion potential of liver cancer cells through Snail. METTL3 increased the m^6^A level in the coding sequence (CDS) of Snail which was recognized by YTHDF1 thus triggering the polysome-mediated translation of Snail mRNA [72]. In HCC cells METTL3 was found SUMOylated by a small ubiquitin-like modifier SUMO1 which increased its expression upon mitogen stimulation. Upregulated METTL3 further regulated the Snail mRNA homeostasis causing HCC progression [73].

## Prospect of m^6^A targeted cancer therapy

While the role of m^6^A modification and its regulators in cancer genesis and progression is becoming increasingly evident with increased research efforts in this field in recent years, the question whether this RNA modification can be targeted for cancer therapeutics is yet to be answered. Since no m^6^A modification-based therapy has been tested so far, it is hard to speculate where we are standing in terms of m^6^A-targeted therapy development. Some efforts have been made in the way of developing m^6^A-targeted therapy. Until now most of the efforts to this end are based mainly on the targeting of m^6^A modifying enzymes. The availability of high resolution crystal structures of these enzymes have facilitated the prediction of potential binding sites which may be utilized to design molecules with high affinity [103]. An in silico method was used to develop ligands which may bind specifically to the m^6^A methylation writer complex (METTL3/METTL14/WTAP) with high affinity and the ligands were tested for their activity and kinetics of binding toward these enzymes [104].

The demethylation activity of FTO and ALKBH5 rely on Fe^2+^, 2-oxoglutarate (2OG) and O_2_. Inhibitors of 2OG or molecules that interfere with the Fe^2+^ binding to the demethylases may act as m^6^A demethylase inhibitors. Niu et al. compiled the list of such compounds that have been tested for their demethylase inhibitor activity, but unfortunately none of the candidate molecules showed selectivity therefore could not be used for therapeutic purposes [105]. The natural compound ‘Rhein’ showed some degree of selectivity with low toxic effect and meclofenamic acid (MA), a non-steroidal anti-inflammatory agent showed potent inhibitory effect against FTO which exhibited promises as demethylase inhibitors. MA2, an ethyl ester derivative of MA is hydrolyzed inside the cell to yield its active form MA. With in vitro experiments MA2 was shown to increase the m^6^A modification level in HeLa cells and had better cellular penetrability than MA [105, 106]. However, none of these were tested clinically for their effectiveness.

## Conclusion and perspectives

m^6^A regulators may act directly on the key signaling molecules causing their up/downregulation by changing their m^6^A status or indirectly via acting on and changing the expression levels of other proteins associated with the pathway. For example- METTL3 overexpression contributed to Wnt/β-catenin pathway activation and hepatoblastoma (HB) tumor progression via direct upregulation of Wnt/β-catenin pathway-associated proteins such as- β-catenin, etc. or indirectly via increasing m^6^A level and upregulation of CTNNB1gene encoding β-catenin [29, 30]. m^6^A modification has also been shown to simultaneously activate more than one signaling pathways in carcinogenesis. METTL3 has been found to play a protumorigenic role in HCC through suppressing both p53 and Ras/Raf/ERK signaling by acting on a common target that regulates both of these pathways [53].

**Fig. 3:**
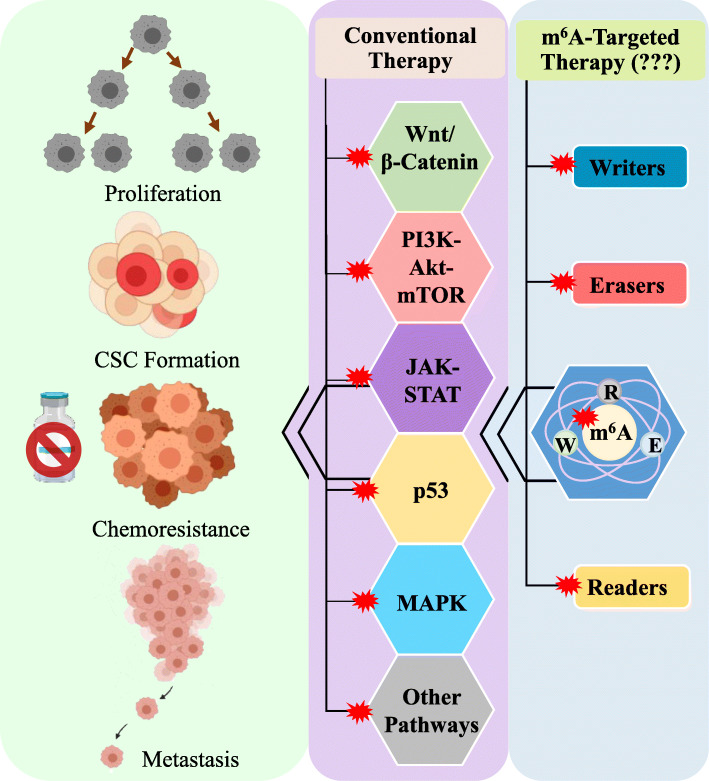
Prospect of targeting m^6^A regulators in developing novel cancer therapeutics. m^6^A RNA modification contributes to tumor progression and/or recurrence by modulating multiple cellular oncogenic signaling pathways. Targeting m^6^A regulatory proteins with selective molecules may interfere with the expression of m^6^A regulatory proteins changing the m^6^A status of the target signaling molecules there by blocking or activating the oncogenic or tumor-suppressive signaling pathways which may stem cancer progression or tame the therapeutically refractory tumors. CSC: Cancer Stem Cell

While significant progresses have been made in understanding important roles of m^6^A modification in various oncogenic signaling pathways as discussed in this review, further studies are needed to strengthen the link between m^6^A modification dysregulation and cancer to establish m^6^A regulators as effective cancer therapeutic targets. First, m^6^A modification may play dual roles in cancer, being oncogenic in some cases while tumor suppressive in others. In human osteosarcoma (OS), upregulation of METTL3 increased tumor progression by activating Wnt/β-catenin pathway while in renal cell carcinoma (RCC), lower METTL3 expression was associated with increased tumor size and advancement due to PI3K-AKT-mTOR pathway activation [32, 42]. Why the same molecule plays opposite roles needs further insight. This seemingly contrasting phenomena was also observed in similar cancer types as in the case of colorectal cancer (CRC). In one instance, increased METTL3 expression was correlated with decreased tumor size, advancement and metastasis, while in the other METTL3 was found to promote CRC metastasis. One explanation for these discrepant results may be the variability in the distribution of patient populations based on METTL3 expression. In the latter case the populations were evenly distributed between high and low expression groups, while in the former case there was a high variability between these two groups which may have contributed to the variable outcome of the analysis. These discrepancies may also be due to differences in signaling molecules targeted by METTL3, either activating or suppressing the MAPK pathway [51, 52]. Similar discrepancy was also observed in case of gastric cancer (GC) where METTL3 downregulation was correlated with malignant phenotype in one case while the opposite was observed in the other. Again, this may be due to variable population size or differences in the molecules targeted by METTL3 thereby activating two distinct pathways [36, 54]. Second, current studies on the role of m^6^A modification in cancer are mostly carried out in cancer cells, whether m^6^A modification dysregulation plays a role in the process of converting normal cells to cancer cells remains largely unknown [107]. Further studies are needed to determine whether and how m^6^A modification dysregulation contributes to cell malignant transformation caused by various cancer etiology factors. Third, it is now known that m^6^A modifications are present in different types of cellular RNAs (e.g. mRNAs, lncRNAs, miRNAs etc.). However, current studies on the role of m^6^A in modulating oncogenic signaling pathways focus mainly on mRNAs. Further studies are needed to determine the role of m^6^A dysregulation in non-coding RNAs especially the lncRNAs in regulating oncogenic signaling pathways in cancer initiation and progression. Fourth, m^6^A modification affects signaling molecules in more than one pathway simultaneously, it is highly likely that altered expression of a single m^6^A regulator may play key roles in dysregulating multiple oncogenic pathways. Therefore, targeting a single m^6^A regulatory molecule may lead to inhibition of multiple oncogenic signaling pathways (Fig. 3). Further studies are needed to determine whether targeting m^6^A regulators alone or in combination with other currently approved anticancer drugs could be effective cancer therapeutic strategies.

## Data Availability

Not applicable

## Abbreviations

5′-UTRs: 5′ untranslated regions
3′-UTRs: 3′ untranslated regions
SAM: S-adenosylmethionine
A: Adenosine
METTL3: Methyltransferase like-3
METTL14: Methyltransferase like-14
WTAP: Wilms’ tumour 1-associating protein
RBM15: RNA-binding motif protein
VIRMA: Vir-like m^6^A methyltransferase associated protein
ZC3H13: Zinc finger CCCH domain-containing protein13
FTO: Fat mass and obesity-associated protein
YTHDF: YTH domain-containing family proteins
RBMs: RNA-binding motifs
lncRNA: Long non-coding RNA
hnRNPC: Heterogeneous nuclear ribonucleoprotein C
eIF3: Eukaryotic initiation factor 3
IGF2BPs: Insulin-like growth factor-2 mRNA binding proteins
HuR: Hu antigen R
NXF1: Nuclear RNA export factor 1
Fz: Frizzled
LRP: Low-density lipoprotein (LDL) receptor-related protein
Dvl: Dishevelled
GSK-3: Glycogen synthase kinase-3
APC: Adenomatous Polyposis Coli
HB: Hepatoblastoma
PARPi: PARP inhibitor
EOC: Epithelial ovarian cancer
OS: Osteosarcoma
LEF1: Lymphoid enhancer-binding factor 1
WIF1: Wnt inhibitory factor1
CRC: Colorectal cancer
PI3Ks: Phosphatidylinositide 3-kinases
PKB: Protein kinase B
mTOR: Mammalian target of rapamycin
PIs: Phosphatidylinositides
PI-4,5-P2: Phosphatidylinositol-(4,5)-bisphosphate
PI3,4,5-P3: Phosphatidylinositol-(3,4,5)-trisphosphate
PDK1: PI3K-dependent kinase-1
GSK3: Glycogen synthase kinase 3
IKK: IκB kinase
TSC2: Tuberous sclerosis 2
VEGFRs: Vascular endothelial growth factor receptors
mTORC1: mTOR complex1
mTORC2: mTOR complex2
GC: Gastric cancer
GI: Gastrointestinal
SAH: S-adenosylhomocysteine
MA: Meclofenamic acid
NPC: Nasopharyngeal carcinoma
IGF1R: Insulin-like growth factor 1 receptor
RCC: Renal cell carcinoma
JAK: Janus kinase
STAT: Signal transducer and activator of transcription
SOCS: Suppressors of cytokine signaling
PIAS: Protein inhibitors of activated stats
PTPs: Protein tyrosine phosphatases
HCC: Hepatocellular carcinoma
LGR5: Leucine-rich repeat-containing G protein-coupled receptor 5
MAPK: Mitogen-activated protein kinase
MAPKKK: MAPK kinase kinase
MAPKK: MAPK kinase
ERK: Extracellular signal-regulated kinase
JNK: c-Jun N-terminal kinase
BATF2: Basic leucine zipper ATF-like transcription factor 2
HGSOC: High-grade serous ovarian carcinoma
ATM: Ataxia telangiectasia mutated
ATR: Ataxia telangiectasia related
AML: Acute myeloid leukemia
ccRCC: Clear cell renal cell carcinoma
ESCA: Esophageal carcinoma
GEMs: Glycosphingolipid-enriched microdomains
GCS: Glucosylceramide synthase
NSCLC: Non-small cell lung cancer
USP7: Ubiquitin-specific protease-7
MST1/2: Mammalian Ste20-like kinases 1/2
LATS1/2: Large tumor suppressor 1/2
Sav1: Salvador homolog 1
MOBKL: MOB kinase activator
YAP: Yes association protein
TEAD: TEA-domain-containing
AGO2: Argonaute RISC catalytic component 2
LUAD: Lung adenocarcinoma
SUV39H1: Suppressor of variegation 39 homolog 1
ERRγ: Estrogen receptor related receptors γ
BCa: Bladder cancer
CDS: Coding sequence
2OG: 2-oxoglutarate

## References

[1] Saletore Y, Meyer K, Korlach J, Vilfan ID, Jaffrey S, Mason CE (2012). The birth of the Epitranscriptome: deciphering the function of RNA modifications. Genome Biol.

[2] He C (2010). Grand challenge commentary: RNA epigenetics?. Nat Chem Biol.

[3] Yu S, Li X, Liu S, Yang R, Liu X, Wu S. N^6^-methyladenosine: a novel RNA imprint in human cancer. Front Oncol. 2019;9. 10.3389/fonc.2019.01407. 10.3389/fonc.2019.01407 PMC6930912 31921664

[4] Balacco DL, Soller M (2018). The m^6^A writer: rise of a machine for growing tasks. Biochemistry..

[5] Uddin MB, Wang Z, Yang C (2020). Dysregulations of functional RNA modifications in cancer, cancer stemness and cancer therapeutics. Theranostics..

[6] Bokar JA, Rath-Shambaugh ME, Ludwiczak R, Narayan P, Rottman F (1994). Characterization and partial purification of mRNA N6-adenosine methyltransferase from HeLa cell nuclei. Internal mRNA methylation requires a multisubunit complex. J Biol Chem.

[7] Liu J, Yue Y, Han D, Wang X, Fu Y, Zhang L, Jia G, Yu M, Lu Z, Deng X, Dai Q, Chen W, He C (2014). A METTL3–METTL14 complex mediates mammalian nuclear RNA N^6^-adenosine methylation. Nat Chem Biol.

[8] Ping X-L, Sun B-F, Wang L, Xiao W, Yang X, Wang W-J, Adhikari S, Shi Y, Lv Y, Chen YS, Zhao X, Li A, Yang Y, Dahal U, Lou XM, Liu X, Huang J, Yuan WP, Zhu XF, Cheng T, Zhao YL, Wang X, Danielsen JMR, Liu F, Yang YG (2014). Mammalian WTAP is a regulatory subunit of the RNA N6-methyladenosine methyltransferase. Cell Res.

[9] Patil DP, Chen C-K, Pickering BF, Chow A, Jackson C, Guttman M, Jaffrey SR (2016). m^6^A RNA methylation promotes XIST-mediated transcriptional repression. Nature..

[10] Yue Y, Liu J, Cui X, Cao J, Luo G, Zhang Z (2018). VIRMA mediates preferential m^6^A mRNA methylation in 3′UTR and near stop codon and associates with alternative polyadenylation. Cell Discov.

[11] Warda AS, Kretschmer J, Hackert P, Lenz C, Urlaub H, Höbartner C, Sloan KE, Bohnsack MT (2017). Human METTL16 is a N^6^-methyladenosine (m^6^A) methyltransferase that targets pre-mRNAs and various non-coding RNAs. EMBO Rep.

[12] Liu N, Pan T (2015). RNA epigenetics. Transl Res.

[13] Jia G, Fu Y, Zhao X, Dai Q, Zheng G, Yang Y, Yi C, Lindahl T, Pan T, Yang YG, He C (2011). N6-methyladenosine in nuclear RNA is a major substrate of the obesity-associated FTO. Nat Chem Biol.

[14] Fu Y, Jia G, Pang X, Wang RN, Wang X, Li CJ (2013). FTO-mediated formation of N^6^-hydroxymethyladenosine and N^6^-formyladenosine in mammalian RNA. Nat Commun.

[15] Yue Y, Liu J, He C (2015). RNA N^6^-methyladenosine methylation in post-transcriptional gene expression regulation. Genes Dev.

[16] Yang G, Sun Z, Zhang N (2020). Reshaping the role of m6A modification in cancer transcriptome: a review. Cancer Cell Int.

[17] Ueda Y, Ooshio I, Fusamae Y, Kitae K, Kawaguchi M, Jingushi K, Hase H, Harada K, Hirata K, Tsujikawa K (2017). AlkB homolog 3-mediated tRNA demethylation promotes protein synthesis in cancer cells. Sci Rep.

[18] Dominissini D, Moshitch-Moshkovitz S, Schwartz S, Salmon-Divon M, Ungar L, Osenberg S, Cesarkas K, Jacob-Hirsch J, Amariglio N, Kupiec M, Sorek R, Rechavi G (2012). Topology of the human and mouse m^6^A RNA methylomes revealed by m^6^A-seq. Nature..

[19] Xu C, Wang X, Liu K, Roundtree IA, Tempel W, Li Y, Lu Z, He C, Min J (2014). Structural basis for selective binding of m^6^A RNA by the YTHDC1 YTH domain. Nat Chem Biol.

[20] Hsu PJ, Zhu Y, Ma H, Guo Y, Shi X, Liu Y, Qi M, Lu Z, Shi H, Wang J, Cheng Y, Luo G, Dai Q, Liu M, Guo X, Sha J, Shen B, He C (2017). Ythdc2 is an N^6^-methyladenosine binding protein that regulates mammalian spermatogenesis. Cell Res.

[21] Liu N, Dai Q, Zheng G, He C, Parisien M, Pan T (2015). N^6^-methyladenosine-dependent RNA structural switches regulate RNA-protein interactions. Nature..

[22] Alarcón CR, Goodarzi H, Lee H, Liu X, Tavazoie S, Tavazoie SF (2015). HNRNPA2B1 is a mediator of m^6^A-dependent nuclear RNA processing events. Cell..

[23] Meyer KD, Patil DP, Zhou J, Zinoviev A, Skabkin MA, Elemento O, Pestova TV, Qian SB, Jaffrey SR (2015). 5′ UTR m^6^A promotes cap-independent translation. Cell..

[24] Huang H, Weng H, Sun W, Qin X, Shi H, Wu H, Zhao BS, Mesquita A, Liu C, Yuan CL, Hu YC, Hüttelmaier S, Skibbe JR, Su R, Deng X, Dong L, Sun M, Li C, Nachtergaele S, Wang Y, Hu C, Ferchen K, Greis KD, Jiang X, Wei M, Qu L, Guan JL, He C, Yang J, Chen J (2018). Recognition of RNA N^6^-methyladenosine by IGF2BP proteins enhances mRNA stability and translation. Nat Cell Biol.

[25] Lewis CJ, Pan T, Kalsotra A (2017). RNA modifications and structures cooperate to guide RNA-protein interactions. Nat Rev Mol Cell Biol.

[26] Liu N, Pan T (2016). N^6^-methyladenosine-encoded epitranscriptomics. Nat Struct Mol Biol.

[27] Delaunay S, Frye M (2019). RNA modifications regulating cell fate in cancer. Nat Cell Biol.

[28] Zhou KI, Pan T (2018). An additional class of m^6^A readers. Nat Cell Biol.

[29] Cui X, Wang Z, Li J, Zhu J, Ren Z, Zhang D (2020). Cross talk between RNA N6-methyladenosine methyltransferase-like 3 and miR-186 regulates hepatoblastoma progression through Wnt/β-catenin signalling pathway. Cell Prolif.

[30] Liu L, Wang J, Sun G, Wu Q, Ma J, Zhang X (2019). m^6^A mRNA methylation regulates CTNNB1 to promote the proliferation of hepatoblastoma. Mol Cancer.

[31] Fukumoto T, Zhu H, Nacarelli T, Karakashev S, Fatkhutdinov N, Wu S, Liu P, Kossenkov AV, Showe LC, Jean S, Zhang L, Zhang R (2019). N^6^-methylation of adenosine of FZD10 mRNA contributes to PARP inhibitor resistance. Cancer Res.

[32] Miao W, Chen J, Jia L, Ma J, Song D (2019). The m6A methyltransferase METTL3 promotes osteosarcoma progression by regulating the m6A level of LEF1. Biochem Biophys Res Commun.

[33] Tang B, Yang Y, Kang M, Wang Y, Wang Y, Bi Y (2020). m^6^A demethylase ALKBH5 inhibits pancreatic cancer tumorigenesis by decreasing WIF-1 RNA methylation and mediating Wnt signaling. Mol Cancer.

[34] Bai Y, Yang C, Wu R, Huang L, Song S, Li W, Yan P, Lin C, Li D, Zhang Y (2019). YTHDF1 regulates Tumorigenicity and Cancer stem cell-like activity in human colorectal carcinoma. Front Oncol.

[35] Pi J, Wang W, Ji M, Wang X, Wei X, Jin J, et al. YTHDF1 promotes gastric carcinogenesis by controlling translation of FZD7. Cancer Res. 2020:canres.0066.2020. 10.1158/0008-5472.CAN-20-0066. 10.1158/0008-5472.CAN-20-0066 32788173

[36] Zhang C, Zhang M, Ge S, Huang W, Lin X, Gao J, Gong J, Shen L (2019). Reduced m6A modification predicts malignant phenotypes and augmented Wnt/PI3K-Akt signaling in gastric cancer. Cancer Med.

[37] Liu J, Eckert MA, Harada BT, Liu S-M, Lu Z, Yu K, Tienda SM, Chryplewicz A, Zhu AC, Yang Y, Huang JT, Chen SM, Xu ZG, Leng XH, Yu XC, Cao J, Zhang Z, Liu J, Lengyel E, He C (2018). m6A mRNA methylation regulates AKT activity to promote the proliferation and tumorigenicity of endometrial cancer. Nat Cell Biol.

[38] Zhang Z, Zhou D, Lai Y, Liu Y, Tao X, Wang Q, Zhao G, Gu H, Liao H, Zhu Y, Xi X, Feng Y (2012). Estrogen induces endometrial cancer cell proliferation and invasion by regulating the fat mass and obesity-associated gene via PI3K/AKT and MAPK signaling pathways. Cancer Lett.

[39] Bi X, Lv X, Liu D, Guo H, Yao G, Wang L, et al. METTL3-mediated maturation of miR-126-5p promotes ovarian cancer progression via PTEN-mediated PI3K/Akt/mTOR pathway. Cancer Gene Ther. 2020:1–15. 10.1038/s41417-020-00222-3 32939058

[40] Zhao Q, Zhao Y, Hu W, Zhang Y, Wu X, Lu J, Li M, Li W, Wu W, Wang J, du F, Ji H, Yang X, Xu Z, Wan L, Wen Q, Li X, Cho CH, Zou C, Shen J, Xiao Z (2020). m^6^A RNA modification modulates PI3K/Akt/mTOR signal pathway in gastrointestinal cancer. Theranostics..

[41] He J, Li Z, Zhuoxian R, Gao J, Mu Y, Guan Y-D (2020). m^6^A reader YTHDC2 promotes radiotherapy resistance of nasopharyngeal carcinoma via activating IGF1R/AKT/S6 signaling axis. Front Oncol.

[42] Li X, Tang J, Huang W, Wang F, Li P, Qin C, Qin Z, Zou Q, Wei J, Hua L, Yang H, Wang Z (2017). The M6A methyltransferase METTL3: acting as a tumor suppressor in renal cell carcinoma. Oncotarget..

[43] Shi X, Ran L, Liu Y, Zhong SH, Zhou PP, Liao MX (2018). Knockdown of hnRNP A2/B1 inhibits cell proliferation, invasion and cell cycle triggering apoptosis in cervical cancer via PI3K/AKT signaling pathway. Oncol Rep.

[44] Liu Y, Wang R, Zhang L, Li J, Lou K, Shi B (2017). The lipid metabolism gene FTO influences breast cancer cell energy metabolism via the PI3K/AKT signaling pathway. Oncol Lett.

[45] Yu H-L, Ma X-D, Tong J-F, Li J-Q, Guan X-J, Yang J-H (2019). WTAP is a prognostic marker of high-grade serous ovarian cancer and regulates the progression of ovarian cancer cells. OncoTargets Ther.

[46] Chen J, Sun Y, Xu X, Wang D, He J, Zhou H, Lu Y, Zeng J, du F, Gong A, Xu M (2017). YTH domain family 2 orchestrates epithelial-mesenchymal transition/proliferation dichotomy in pancreatic cancer cells. Cell Cycle.

[47] Chen M, Wei L, Law CT, Tsang FHC, Shen J, Cheng CLH, Tsang LH, Ho DWH, Chiu DKC, Lee JMF, Wong CCL, Ng IOL, Wong CM (2018). RNA N6-methyladenosine methyltransferase-like 3 promotes liver cancer progression through YTHDF2-dependent posttranscriptional silencing of SOCS2. Hepatology..

[48] Xu J, Chen Q, Tian K, Liang R, Chen T, Gong A, Mathy N, Yu T, Chen X (2020). m6A methyltransferase METTL3 maintains colon cancer tumorigenicity by suppressing SOCS2 to promote cell proliferation. Oncol Rep.

[49] Jiang L, Chen T, Xiong L, Xu JH, Gong AY, Dai B (2020). Knockdown of m6A methyltransferase METTL3 in gastric cancer cells results in suppression of cell proliferation. Oncol Lett.

[50] Gong D, Zhang J, Chen Y, Xu Y, Ma J, Hu G, Huang Y, Zheng J, Zhai W, Xue W (2019). The m^6^A-suppressed P2RX6 activation promotes renal cancer cells migration and invasion through ATP-induced Ca 2+ influx modulating ERK1/2 phosphorylation and MMP9 signaling pathway. J Exp Clin Cancer Res.

[51] Deng R, Cheng Y, Ye S, Zhang J, Huang R, Li P, Liu H, Deng Q, Wu X, Lan P, Deng Y (2019). m^6^A methyltransferase METTL3 suppresses colorectal cancer proliferation and migration through p38/ERK pathways. OncoTargets Ther.

[52] Peng W, Li J, Chen R, Gu Q, Yang P, Qian W, Ji D, Wang Q, Zhang Z, Tang J, Sun Y (2019). Upregulated METTL3 promotes metastasis of colorectal cancer via miR-1246/SPRED2/MAPK signaling pathway. J Exp Clin Cancer Res.

[53] Chen SL, Liu LL, Wang CH, Lu SX, Yang X, He YF, Zhang CZ, Yun JP (2020). Loss of RDM1 enhances hepatocellular carcinoma progression via p53 and Ras/Raf/ERK pathways. Mol Oncol.

[54] Xie J-W, Huang X-B, Chen Q-Y, Ma Y-B, Zhao Y-J, Liu L-C (2020). m^6^A modification-mediated BATF2 acts as a tumor suppressor in gastric cancer through inhibition of ERK signaling. Mol Cancer.

[55] Yu T, Xu Y, Sun T, Huang D, Cao F, Gao X (2020). YTHDC2 promotes the apoptosis of colorectal cancer cells through the p38MAPK signaling pathway.

[56] Zhong L, Liao D, Zhang M, Zeng C, Li X, Zhang R, Ma H, Kang T (2019). YTHDF2 suppresses cell proliferation and growth via destabilizing the EGFR mRNA in hepatocellular carcinoma. Cancer Lett.

[57] Guo X, Li K, Jiang W, Hu Y, Xiao W, Huang Y (2020). RNA demethylase ALKBH5 prevents pancreatic cancer progression by posttranscriptional activation of PER1 in an m^6^A-YTHDF2-dependent manner. Mol Cancer.

[58] Zhou J, Wang J, Hong B, Ma K, Xie H, Li L (2019). Gene signatures and prognostic values of m6A regulators in clear cell renal cell carcinoma–a retrospective study using TCGA database. Aging (Albany NY).

[59] Yang W, Duan L, Zhao X, Wang X, Li Y, Niu L (2020). Identification and validation of a novel prognostic index based on m6A RNA methylation regulators in esophageal carcinoma.

[60] Uddin MB, Roy KR, Hosain SB, Khiste SK, Hill RA, Jois SD, Zhao Y, Tackett AJ, Liu YY (2019). An N^6^-methyladenosine at the transited codon 273 of p53 pre-mRNA promotes the expression of R273H mutant protein and drug resistance of cancer cells. Biochem Pharmacol.

[61] Li J, Han Y, Zhang H, Qian Z, Jia W, Gao Y, Zheng H, Li B (2019). The m6A demethylase FTO promotes the growth of lung cancer cells by regulating the m6A level of USP7 mRNA. Biochem Biophys Res Commun.

[62] Jin D, Guo J, Wu Y, Du J, Yang L, Wang X (2019). m^6^A mRNA methylation initiated by METTL3 directly promotes YAP translation and increases YAP activity by regulating the MALAT1-miR-1914-3p-YAP axis to induce NSCLC drug resistance and metastasis. J Hematol Oncol.

[63] Jin D, Guo J, Wu Y, Yang L, Wang X, Du J (2020). m^6^A demethylase ALKBH5 inhibits tumor growth and metastasis by reducing YTHDFs-mediated YAP expression and inhibiting miR-107/LATS2–mediated YAP activity in NSCLC. Mol Cancer.

[64] Lin S, Choe J, Du P, Triboulet R, Gregory RI (2016). The m^6^A methyltransferase METTL3 promotes translation in human cancer cells. Mol Cell.

[65] Chen X, Xu M, Xu X, Zeng K, Liu X, Sun L, Pan B, He B, Pan Y, Sun H, Xia X, Wang S (2020). METTL14 suppresses CRC progression via regulating N6-Methyladenosine-dependent primary miR-375 processing. Mol Ther.

[66] Zheng Q, Lin Z, Li X, Xin X, Wu M, An J, Gui X, Li T, Pu H, Li H, Lu D (2016). Inflammatory cytokine IL6 cooperates with CUDR to aggravate hepatocyte-like stem cells malignant transformation through NF-κB signaling. Sci Rep.

[67] Chen Z, Wu L, Zhou J, Lin X, Peng Y, Ge L, Chiang CM, Huang H, Wang H, He W (2020). N6-methyladenosine-induced ERRγ triggers chemoresistance of cancer cells through upregulation of ABCB1 and metabolic reprogramming. Theranostics..

[68] Jiang Y, Wan Y, Gong M, Zhou S, Qiu J, Cheng W (2020). RNA demethylase ALKBH5 promotes ovarian carcinogenesis in a simulated tumour microenvironment through stimulating NF-κB pathway. J Cell Mol Med.

[69] Cheng M, Sheng L, Gao Q, Xiong Q, Zhang H, Wu M, Liang Y, Zhu F, Zhang Y, Zhang X, Yuan Q, Li Y (2019). The m^6^A methyltransferase METTL3 promotes bladder cancer progression via AFF4/NF-κB/MYC signaling network. Oncogene..

[70] Cai J, Yang F, Zhan H, Situ J, Li W, Mao Y, Luo Y (2019). RNA m^6^A methyltransferase METTL3 promotes the growth of prostate cancer by regulating hedgehog pathway. OncoTargets Ther.

[71] Visvanathan A, Patil V, Abdulla S, Hoheisel JD, Somasundaram K (2019). N^6^-Methyladenosine landscape of glioma stem-like cells: METTL3 is essential for the expression of actively transcribed genes and sustenance of the oncogenic signaling. Genes..

[72] Lin X, Chai G, Wu Y, Li J, Chen F, Liu J (2019). RNA m^6^A methylation regulates the epithelial mesenchymal transition of cancer cells and translation of snail. Nat Commun.

[73] Xu H, Wang H, Zhao W, Fu S, Li Y, Ni W, Xin Y, Li W, Yang C, Bai Y, Zhan M, Lu L (2020). SUMO1 modification of methyltransferase-like 3 promotes tumor progression via regulating snail mRNA homeostasis in hepatocellular carcinoma. Theranostics..

[74] Clevers H, Nusse R (2012). Wnt/β-catenin signaling and disease. Cell..

[75] Logan CY, Nusse R (2004). The Wnt signaling pathway in development and disease. Annu Rev Cell Dev Biol.

[76] Pai SG, Carneiro BA, Mota JM, Costa R, Leite CA, Barroso-Sousa R, Kaplan JB, Chae YK, Giles FJ (2017). Wnt/beta-catenin pathway: modulating anticancer immune response. J Hematol Oncol.

[77] MacDonald BT, Tamai K, He X (2009). Wnt/β-catenin signaling: components, mechanisms, and diseases. Dev Cell.

[78] Chamcheu JC, Roy T, Uddin MB, Banang-Mbeumi S, Chamcheu R-CN, Walker AL, Liu YY, Huang S (2019). Role and therapeutic targeting of the PI3K/Akt/mTOR signaling pathway in skin cancer: a review of current status and future trends on natural and synthetic agents therapy. Cells..

[79] Jason S, Cui W (2016). Proliferation, survival and metabolism: the role of PI3K/AKT/mTOR signalling in pluripotency and cell fate determination. Development..

[80] Porta C, Paglino C, Mosca A (2014). Targeting PI3K/Akt/mTOR signaling in cancer. Front Oncol.

[81] Saxton RA, Sabatini DM (2017). mTOR signaling in growth, metabolism, and disease. Cell..

[82] Rawlings JS, Rosler KM, Harrison DA (2004). The JAK/STAT signaling pathway. J Cell Sci.

[83] Li WX (2008). Canonical and non-canonical JAK–STAT signaling. Trends Cell Biol.

[84] Quintás-Cardama A, Verstovsek S (2013). Molecular pathways: Jak/STAT pathway: mutations, inhibitors, and resistance. Clin Cancer Res.

[85] Morris R, Kershaw NJ, Babon JJ (2018). The molecular details of cytokine signaling via the JAK/STAT pathway. Protein Sci.

[86] Luo N, Balko JM (2019). Role of JAK-STAT pathway in cancer signaling. Predictive biomarkers in oncology.

[87] Li H-B, Tong J, Zhu S, Batista PJ, Duffy EE, Zhao J, Bailis W, Cao G, Kroehling L, Chen Y, Wang G, Broughton JP, Chen YG, Kluger Y, Simon MD, Chang HY, Yin Z, Flavell RA (2017). m^6^A mRNA methylation controls T cell homeostasis by targeting the IL-7/STAT5/SOCS pathways. Nature..

[88] Burotto M, Chiou VL, Lee JM, Kohn EC (2014). The MAPK pathway across different malignancies: a new perspective. Cancer..

[89] Dhillon AS, Hagan S, Rath O, Kolch W (2007). MAP kinase signalling pathways in cancer. Oncogene..

[90] Plotnikov A, Zehorai E, Procaccia S, Seger R (2011). The MAPK cascades: signaling components, nuclear roles and mechanisms of nuclear translocation. Biochim Biophys Acta (BBA) Mol Cell Res.

[91] Huang P, Han J, Hui L (2010). MAPK signaling in inflammation-associated cancer development. Protein Cell.

[92] Wagner EF, Nebreda ÁR (2009). Signal integration by JNK and p38 MAPK pathways in cancer development. Nat Rev Cancer.

[93] Bieging KT, Mello SS, Attardi LD (2014). Unravelling mechanisms of p53-mediated tumour suppression. Nat Rev Cancer.

[94] Brooks CL, Gu W (2006). p53 ubiquitination: Mdm2 and beyond. Mol Cell.

[95] Vogelstein B, Lane D, Levine AJ (2000). Surfing the p53 network. Nature..

[96] Kwok C-T, Marshall AD, Rasko JE, Wong JJ (2017). Genetic alterations of m^6^A regulators predict poorer survival in acute myeloid leukemia. J Hematol Oncol.

[97] Roy KR, Uddin MB, Roy SC, Hill RA, Marshall J, Li YT, Chamcheu JC, Lu H, Liu YY (2020). Gb3-cSrc complex in glycosphingolipid-enriched microdomains contributes to the expression of p53 mutant protein and cancer drug resistance via β-catenin activated RNA-methylation. FASEB BioAdv.

[98] Yu F-X, Guan K-L (2013). The hippo pathway: regulators and regulations. Genes Dev.

[99] Zhao B, Li L, Guan K-L (2010). Hippo signaling at a glance. J Cell Sci.

[100] Piccolo S, Dupont S, Cordenonsi M (2014). The biology of YAP/TAZ: hippo signaling and beyond. Physiol Rev.

[101] Sebio A, Lenz H-J (2015). Molecular pathways: hippo signaling, a critical tumor suppressor. Clin Cancer Res.

[102] Han Y (2019). Analysis of the role of the hippo pathway in cancer. J Transl Med.

[103] Aik W, Demetriades M, Hamdan MK, Bagg EA, Yeoh KK, Lejeune C (2013). Structural basis for inhibition of the fat mass and obesity associated protein (FTO). J Med Chem.

[104] Selberg S, Blokhina D, Aatonen M, Koivisto P, Siltanen A, Mervaala E (2019). Discovery of small molecules that activate RNA methylation through cooperative binding to the METTL3–14-WTAP complex active site. Cell Rep.

[105] Niu Y, Wan A, Lin Z, Lu X, Wan G (2018). N^6^-Methyladenosine modification: a novel pharmacological target for anti-cancer drug development. Acta Pharm Sin B.

[106] Huang Y, Yan J, Li Q, Li J, Gong S, Zhou H, Gan J, Jiang H, Jia GF, Luo C, Yang CG (2015). Meclofenamic acid selectively inhibits FTO demethylation of m^6^A over ALKBH5. Nucleic Acids Res.

[107] Yang C (2020). ToxPoint: dissecting functional RNA modifications in responses to environmental exposure-mechanistic toxicology research enters a new era. Toxicol Sci.

